# miR-98-5p plays a critical role in depression and antidepressant effect of ketamine

**DOI:** 10.1038/s41398-021-01588-0

**Published:** 2021-09-03

**Authors:** Chaoli Huang, Yuanyuan Wang, Zifeng Wu, Jiali Xu, Ling Zhou, Di Wang, Ling Yang, Bin Zhu, Guiquan Chen, Cunming Liu, Chun Yang

**Affiliations:** 1 Department of Anesthesiology and Perioperative Medicine, The First Affiliated Hospital of Nanjing Medical University, Nanjing, 210029 China; 2 State Key Laboratory of Pharmaceutical Biotechnology, Model Animal Research Center, Nanjing University, Nanjing, 210061 China; 3 Department of Cardiology, The Third Affiliated Hospital of Soochow University, Changzhou, 213003 China; 4 Department of Critical Care Medicine, The Third Affiliated Hospital of Soochow University, Changzhou, 213003 China

**Keywords:** Depression, Predictive markers

## Abstract

Ketamine has been demonstrated to be a rapid-onset and long-lasting antidepressant, but its underlying molecular mechanisms remain unclear. Recent studies have emerged microRNAs as important modulators for depression treatment. In this study, we report that miR-98-5p is downregulated in the prefrontal cortex and hippocampus of mice subjected to chronic social stress, while overexpressing it by its agonist alleviates depression-like behaviors. More importantly, we demonstrate that miR-98-5p is upregulated by ketamine administration, while inhibition of it by its antagonist blocks the antidepressant effect of ketamine. Our data implicate a novel molecular mechanism underlying the antidepressant effect of ketamine, and that therapeutic strategies targeting miR-98-5p could exert beneficial effects for depression treatment.

## Introduction

Mounting studies demonstrate that ketamine exerts a robust antidepressant effect, especially for refractory depression [1]. The antidepressant effect of ketamine is characterized by a rapid onset of action within hours and a long-lasting effect up to 2 weeks after a single dose [2, 3], attracting increasing scientific attentions and concerns. However, despite its clinical efficacy, the detailed mechanisms of ketamine exerting facilitating effects for depression are still obscure. Recent studies propose that in addition to its ability to inhibit N-methyl-D-aspartate (NMDA) receptors, the antidepressant effect of ketamine might involve epigenetic mechanisms, such as microRNA (miRNA) regulation [4].

miRNAs are a subset of endogenous small noncoding RNA molecules that serve as post-transcriptional regulators. It is well elucidated that miRNAs are associated with the pathophysiology and therapeutic mechanisms of depression [5]. Several miRNAs have been reported to be involved in pathogenesis and response to treatment of depression, and could act as potential targets for novel antidepressant treatment [6–9]. Very recently, the antidepressant effect of ketamine is closely related to the regulation of various miRNAs in the brain [10, 11].

In the present study, we adopted microarray analysis to assess miRNA expressions in the prefrontal cortex (PFC) of resilient and susceptible phenotypes in chronic social defeat stress (CSDS) mice, and 3 miRNAs were selected as potential candidates in the regulation of depression. We demonstrated that interference of these miRNAs attenuated depression-like behaviors in CSDS-susceptible mice. More importantly, we found that an antidepressant dose of ketamine leaded to an increased expression of miR-98-5p, and inhibition of miR-98-5p diminished the antidepressant effect of ketamine in CSDS mouse model.

## Materials and methods

### Animals

Male adult C57BL/6 mice aged eight weeks (body weight 20–25 g) and male adult ICR mice aged 13–15 weeks (body weight 40–50 g) were purchased from GemPharmatech (Nanjing, China). Animals were maintained in a specific pathogen-free (SPF) level of animal room in the facility of the Model Animal Research Center (MARC) of Nanjing University. The mice were kept under constant humidity and temperature (25 ± 1 °C) and 12-h light/dark cycles (lights on 7:00 AM–19:00 PM) with *ad libitum* food and water. The protocol was approved by the Nanjing University Institutional Animal Care and Use Committee.

### Chronic social defeat stress paradigm

The procedure of CSDS was performed as previously reported [3, 12–14]. In the CSDS model, 8-week-old C57BL/6 male mice were exposed to a different CD-1 mouse for consecutive 10 days (10 min/day). C57BL/6 J mouse was defeated by a larger CD-1 mouse. After the social defeat session, the defeated mouse was subjected to continuous psychological stress from CD-1 mouse through a clear perforated divider that allows visual, olfactory, and auditory contact in a shared home cage for the remaining 24 h. The C57BL/6 mice were rotated daily, while the CD-1 mice were kept in the same cage during the 10-day defeat procedure. In order to select the susceptible and resilient mice, social-interaction test (SIT) was performed on the next day after the last attack of defeat stress. The SIT was conducted in an interaction-test box (42 × 42 cm) with an empty perforated plastic box (10 × 4.5 cm) located at one end. The 8-cm-wide area surrounding the box was defined as the “interaction zone”. The test was composed of two parts: with and without an unfamiliar CD-1 mouse in the plastic box. The test was performed for 2.5 min for each part and the movements of C57BL/6 mice were recorded. The interaction ratio is a ratio calculated time spent in the interaction zone with a CD-1/time spent in the interaction zone without a CD-1. In general, the mouse, whose interaction ratio was <1, had been regarded as susceptible mouse. The mouse, whose interaction ratio was >1, was classified as resilient. In our experiment, approximately 25% of mice were resilient after CSDS.

### Treatment and behavioral tests

Ketamine (Fujian Gutian Pharmaceutical Company, Fujian, China) was diluted in saline (0.9% [w/v] solution of NaCl) and was administered intraperitoneally (i.p.) at a dosage of 10 mg/kg. The dose was selected as reported previously [3, 15]. The mice in the control group were injected with an equal volume of saline. miRNA agonist, antagonist, negative-control agonist, or negative-control antagonist (RiboBio, Guangzhou, China) was administrated i.p. following the manufacturer’s protocol. Behavioral tests, including locomotion test (LMT), forced-swimming test (FST), and sucrose-preference test (SPT), were performed as we reported previously [3, 12–14].

### Locomotion test

The mice were placed in a wooden box (56 × 56 × 50 cm, length × width × height). The floor of the box was divided into 12 equal squares. The number of squares crossed was counted in a 10 min session.

### Forced-swimming test

The FST was conducted in a cylinder that was 31 cm tall and 23 cm in diameter containing 15 cm of water (23 ± 1 °C). The FST was performed for 6 min, and the immobility time was recorded during the last 5 min. The immobility time was defined as the time during which the mouse stood still without struggling, used only essential movements to keep its head above water.

### Sucrose-preference test

The mice were exposed to water and 1% sucrose solution for 24 h of followed by 24 h water and food deprivation. The mice were allowed to drink one bottle of 1% sucrose solution and another bottle of water freely for 2 h. The water and sucrose consumption were measured, and the sucrose preference was calculated as a percentage of sucrose consumption to the total consumptions of both liquids.

### RNA extraction and real-time quantitative PCR

RNA was extracted from PFC and hippocampus samples using miRNeasy mini kit (QIAGEN) following the manufacturer’s protocol. Reverse transcription was performed using miRNA-specific primers (Applied Biosystems, Foster City, CA, USA). The resulting cDNA was diluted 1:2 prior to performing quantitative real-time PCR (qPCR). qPCR was performed in triplicate using Taqman microRNA probes (Applied Biosystems Inc, CA, USA) following the manufacturer’s protocol.

### miRNA array

PFC samples were collected three days after CSDS and total RNA was isolated with miRNeasy mini kit (QIAGEN) following the manufacturer’s protocol. RNA quality and quantity were determined by Nanodrop spectrophotometer (ThermoFisher Scientific, USA). RNA labeling was conducted using miRCURY™ Hy3™/Hy5™ power labeling kit (Exiqon Inc., Woburn, MA, USA) following the manufacturer’s protocol. After labeling, the RNA was subsequently hybridized on the miRCURY™ LNA Array (v18.0) (Exiqon, Vedbaek, Denmark) following the manufacturer’s protocol. The slides were scanned using Axon GenePix 4000 B microarray scanner (Axon Instruments, Foster City, CA, USA). Scanned images were scanned in GenePix Pro 6.0 software (Axon Instruments, Foster City, CA, USA) for grid alignment and data extraction. miRNAs, whose intensities are more than 50 in all samples, were used to calculate a normalization factor. Expressed data were normalized by median normalization. After normalization, the miRNAs that were significantly differentially expressed were identified through Volcano Plot filtering. Finally, the data were subjected to hierarchical clustering and depicted in a heat-map format using GeneSpring GX software v7.3 (Agilent Technologies, California, United States).

### Statistical analysis

The data were expressed as the mean ± standard error of the mean (SEM). Analyses were performed with GraphPad Prism 8.0 (GraphPad Software Inc, La Jolla, CA, USA). Data in this study were analyzed by one-way, two-way analysis of variance (ANOVA) followed by Tukey post hoc analysis or unpaired *t*-test. *P* < 0.05 was considered significant.

## Results

### Expression of miRNAs in PFC and hippocampus of CSDS-resilient and -susceptible mice

**Fig. 1:**
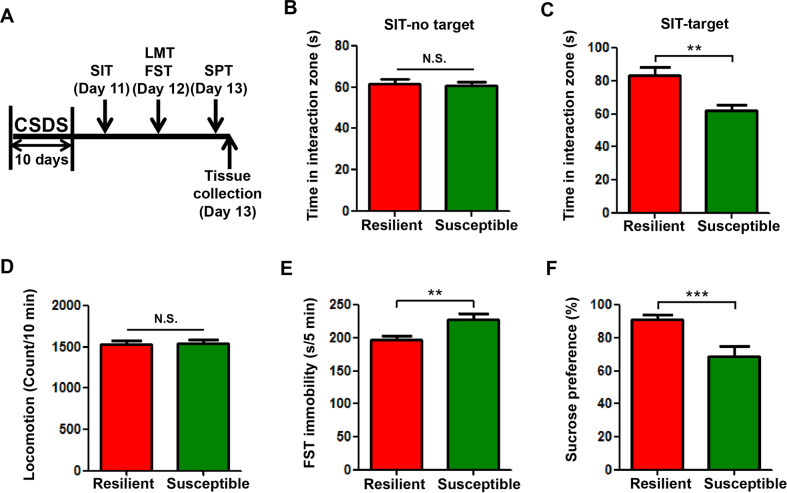
The establishment of SDS mouse model. **a** The schedule of CSDS model and behavioral tests. **b**, **c** The time spent in the interaction zone during the SIT. SIT was performed at day 11 to segregate the defeated mice into resilient and susceptible subgroups. **d**–**f** The number of squares crossed during LMT, immobility time during FST, and sucrose-preference ratio during SPT of CSDS-resilient and -susceptible mice. LMT, FST, and SPT were performed to analyze the depression-like behaviors of CSDS-resilient and -susceptible mice at day 12 and day 13. Data are shown as mean ± SEM (*n* = 8). **P < 0.01, ***P < 0.001, N.S. not significant.

**Fig. 2:**
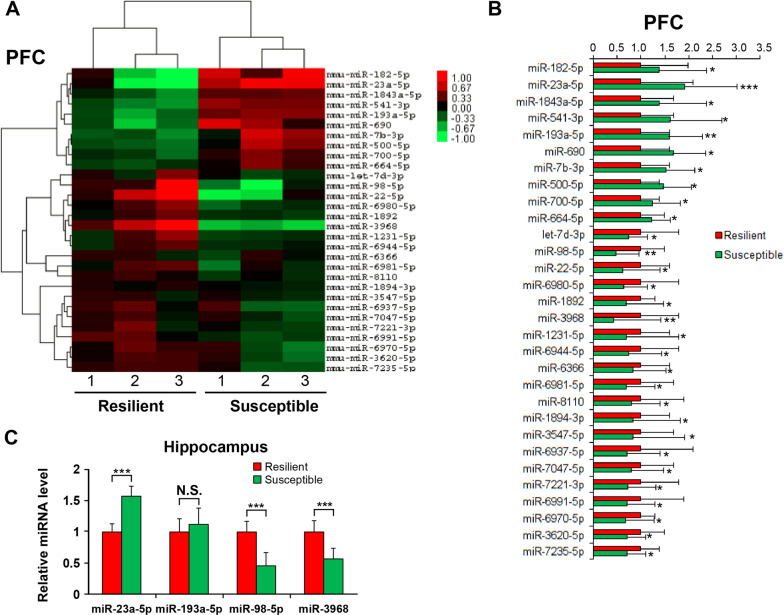
miRNA expressions in PFC and hippocampus of CSDS-resilient and -susceptible mice. **a** Heat map of microarray results. The rows indicate miRNAs and columns indicate samples. The red color represents upregulation and the green color represents downregulation. **b** Relative expression levels of the differentially expressed miRNAs detected in the microarray. **c** Relative expression levels of miR-23a-5p, miR-193a-5p, miR-98-5p, and miR-3968. qPCR was performed to assay the miRNA expressions in the hippocampus of CSDS-resilient and -susceptible mice. Data are shown as mean ± SEM (*n* = 3). *P < 0.05; **P < 0.01; ***P < 0.001; N.S. not significant.

Multiple lines of evidence suggest that miRNAs are associated with the pathogenesis of depression [16]. To examine the effect of CSDS on miRNA expressions in PFC and hippocampus, we first established CSDS mouse model (Fig. 1a–c). After 10 days of CSDS, we carried out behavioral tests and observed that mice in the susceptible group had significantly increased immobility time in the FST, and significantly lowered sucrose-preference ratio in the SPT (Fig. 1d–f). Therefore, the depression model in mice has been successfully established. Subsequently, we collected PFC samples three days after CSDS and conducted miRNA microarray analysis. We identified 30 sets of miRNAs that expressed differentially in the CSDS-susceptible group compared with the resilient group (Fig. 2a), including 10 upregulated miRNAs and 20 downregulated miRNAs (Fig. 2b). In particular, 4 miRNAs (miR-23a-5p, miR-193a-5p, miR-98-5p, and miR-3968) with stable expressions and significant differences between the two groups were selected for further validation according to the *P* value and the FC value.

We next performed qPCR to validate the expressions of the 4 miRNAs in the hippocampus and found that three of them (miR-23a-5p, miR-98-5p, and miR-3968) show significant expression differences (Fig. 2c). These data indicate that miR-23a-5p, miR-98-5p, and miR-3968 may be involved in the pathogenies of depression.

### Effects of miR-23a-5p/-98-5p/-3968 interferences on the depression-like behaviors of CSDS-susceptible mice

**Fig. 3:**
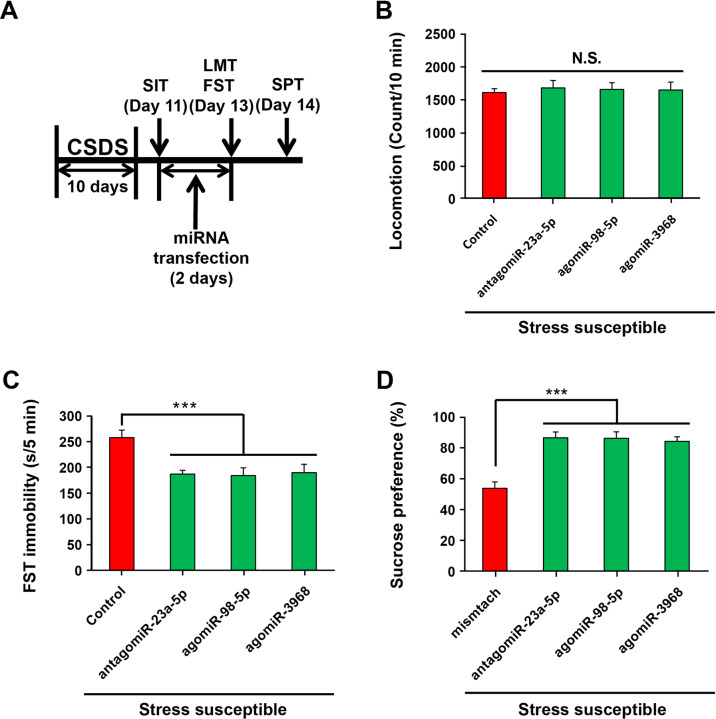
Effects of miR-23a-5p/-98-5p/-3968 inhibition on depression-like behaviors of CSDS-susceptible mice. **a** The schedule of miRNA antagonist or agonist administration and behavioral tests after CSDS. **b**–**d** The number of squares crossed during LMT, immobility time during FST, and sucrose-preference ratio during SPT. LMT, FST, and SPT were performed to analyze the depression-like behaviors of CSDS-susceptible mice after administration with antagonist to miR-23a-5p or agonist to miR-98-5p or miR-3968. Data are shown as mean ± SEM (*n* = 8). ***P < 0.001, N.S. not significant.

To further explore the roles of miR-23a-5p, miR-98-5p, and miR-3968 in the CSDS-induced depression-like behavior, we evaluated the effects of interfering these 3 miRNAs in CSDS-susceptible mice separately. Since miR-23a-5p is significantly upregulated in CSDS-susceptible mice while miR-98-5p and miR-3968 are downregulated, we treated the susceptible mice with antagonist for miR-23a-5p or agonist for miR-98-5p or miR-3968 to interfere with these miRNAs, respectively, and then conducted behavioral tests to examine their depression-like behaviors (Fig. 3a). qPCR was conducted first to verify the efficiency of the miRNA interference (Fig. s1a). The results showed that expressions of miR-23a-5p in PFC and hippocampus were significantly downregulated by the antagonist and expressions of miR-98-5p and miR-3968 were significantly upregulated by the respective agonists (Fig. s1b–d), suggesting that interfering the miRNAs with antagonist or agonist is efficient. Subsequently, the behavioral tests demonstrated that all three groups with miRNA interference had significantly less immobility time in the FST and higher ratio of sucrose preference in the SPT than those in the control group (Fig. 3c, d). Therefore, interference of miR-23a-5p/-98-5p/-3968 could alleviate the depression-like behaviors of CSDS-susceptible mice.

### Effects of ketamine on the expression of miR-23a-5p/-98-5p/-3968 in CSDS-susceptible mice

**Fig. 4:**
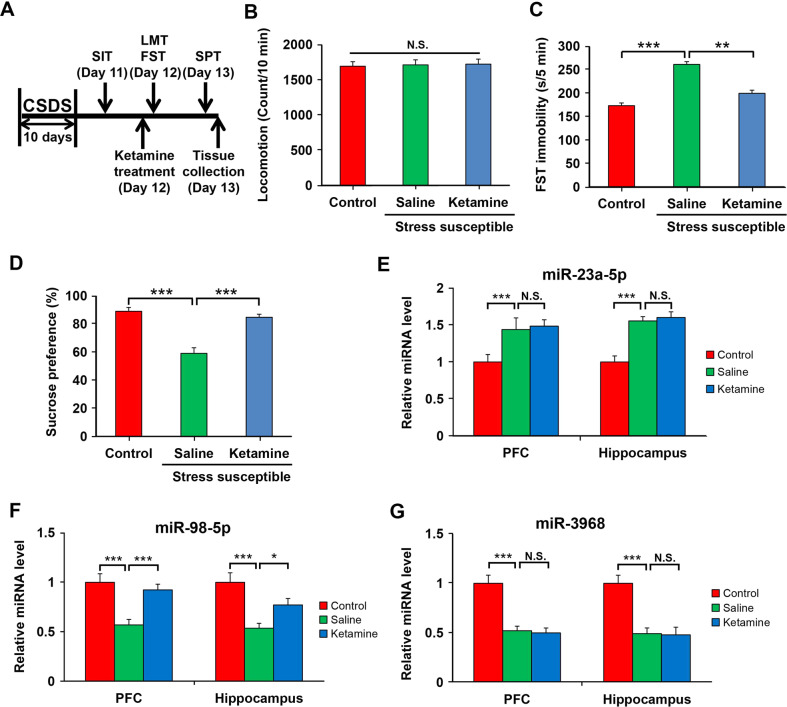
Effects of ketamine on miR-23a-5p/-98-5p/-3968 expression in the PFC and hippocampus of CSDS-susceptible mice. **a** The schedule of ketamine administration and behavioral tests after CSDS. **b**–**d** The number of squares crossed during LMT, immobility time during FST, and sucrose-preference ratio during SPT. LMT, FST, and SPT were performed to analyze the depression-like behaviors of CSDS-resilient and -susceptible mice after ketamine administration. **e**–**g** Relative expression levels of miR-23a-5p, miR-98-5p, and miR-3968. qPCR was performed to assay the miRNA expressions in the PFC and hippocampus of saline-administered control CSDS-resilient mice and saline- or ketamine-administered CSDS-susceptible mice. Data are shown as mean ± SEM (*n* = 8). *P < 0.05, **P < 0.01, ***P < 0.001, N.S. not significant.

Recent studies have linked miRNAs to the antidepressant effect of ketamine [9, 10]. Therefore, we investigated whether the expressions of miR-23a-5p, miR-98-5p, and miR-3968 in PFC and hippocampus respond to ketamine treatment. We first treated CSDS-susceptible mice with an antidepressant dose of ketamine after CSDS, then performed behavioral tests to validate the antidepressant effect of ketamine, and adopted qPCR to analyze the expressions of the 3 miRNAs (Fig. 4a). The behavioral tests illustrated that ketamine markedly decreased the immobility time of the FST and increased sucrose-preference ratio (Fig. 4b–d), confirming the remarkable antidepressant effect of ketamine. qPRC results showed that in both PFC and hippocampus, the levels of miR-98-5p were increased by ketamine treatment, but the levels of miR-23a-5p and miR-3968 were not significantly affected (Fig. 4e–g). These results imply that miR-98-5p is a ketamine-sensitive miRNA, raising the possibility that upregulation of this miRNA may contribute to the antidepressant effect of ketamine.

### Effects of miR-98-5p inhibition on the antidepressant effect of ketamine

**Fig. 5:**
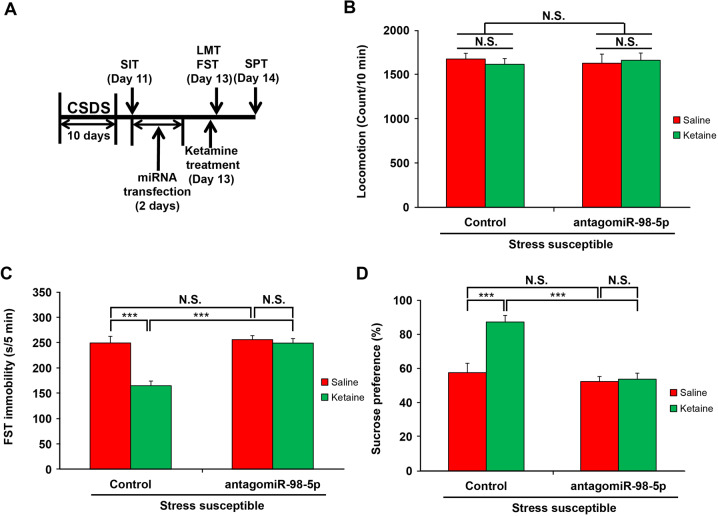
Role of miR-98-5p in the antidepressant mechanism of ketamine. **a** The schedule of miRNA antagonist administration, ketamine administration and behavioral tests after CSDS. **b**–**d** The number of squares crossed during LMT, immobility time during FST and sucrose preference ratio during SPT. LMT, FST, and SPT were performed to analyze the depression-like behaviors of CSDS-susceptible mice after administration with antagonist to miR-98-5p and ketamine. Data are shown as mean ± SEM (*n* = 8). ***P < 0.001, N.S. not significant.

To determine whether the antidepressant effect of ketamine requires upregulation of miR-98-5p, we tested the effects of miR-98-5p on the antidepressant actions of ketamine. We first treated CSDS-susceptible mice with miR-98-5p antagonist and then treated them with ketamine, and finally conducted behavioral tests to examine their depression-like behaviors (Fig. 5a). Although ketamine significantly decreased immobility time in the FST and increased sucrose-preference ratio in the SPT for the control mice, it had no obvious effect on the mice treated with miR-98-5p antagonist (Fig. 5b–d). These results suggest that the antidepressant effect of ketamine is dependent upon the upregulation of miR-98-5p.

## Discussion

miRNAs regulate multiple gene expressions, significantly affect cellular functions, and are implicated in many psychiatric diseases [17]. Growing evidence indicated that miRNAs might play a key role in the pathogenesis of depression [16]. Clinical studies have identified altered expressions of many miRNAs in the peripheral tissues of patients with major depression or bipolar disorder [18, 19], and also have detected aberrant levels of several miRNAs in the brain of patients with major depressive symptoms who died by suicide [18]. Subsequent animal studies replicated miRNAs’ role in depression by interfering some miRNAs to reverse the depression-like behaviors in animal models of depression [20, 21]. To further explore which miRNAs are potentially involved in depression, we took use of CSDS mouse model and profiled the miRNA expressions in the PFC of CSDS-resilient and -susceptible mice by utilizing a microarray technique. Four miRNAs with the most distinctive difference between the two groups were noted and three of them were selected after validating their expressions in the hippocampus, including miR-23a-5p, miR-98-5p, and miR-3968. We interfered their expression in CSDS-susceptible mice by the respective agonist or antagonists and found that reversing the altered expression of any of these miRNAs could drastically reduce the social stress-induced depression-like behaviors. Thus, we implicated 3 miRNAs in the progression of depression. Even though our following studies excluded two of these miRNAs to be critically involved in the mechanism of ketamine’s antidepressant effect, they may still serve as therapeutic targets in depression and worth further investigating.

In recent years, ketamine has become a promising candidate to produce a rapid and sustained antidepressant effect in treatment-resistant patients. Although the antidepressant mechanism of ketamine has not been fully understood, several studies have associated it with regulation of miRNAs [9, 10]. Thus, the 3 miRNAs selected from the expression profile may be involved in the antidepressant mechanism of ketamine. To evaluate this speculation, we tested the effect of ketamine on the expressions of the 3 miRNAs and found that only one of them is significantly affected, which is miR-98-5p. We further interfered this miRNA and successfully blocked the antidepressant effect of ketamine. Collectively, we illustrated that miR-98-5p is involved in the molecular mechanism of ketamine’s antidepressant activity. We speculate that miR-98-5p is also correlated with the side effects of ketamine as an antidepressant. Ketamine can cause extensive cystitis that is characterized by increased urinary frequency and bladder pain [22, 23]. On the other hand, miRNAs have been emerged as important regulators in bladder function and uropathology [24, 25], and abnormal miRNA expressions are considered to be important contributors in bladder inflammation and pain [26]. Thus, the possibility that aberrant expression of miR-98-5p might also account for ketamine-induced uropathy deserves more in-depth study and exploration.

However, this study has some limitations. First, the mechanism by how miR-98-5p is upregulated by ketamine still remains unclear. We speculate that this mechanism may involve the inhibition of glycogen synthase kinase-3 (GSK3). This is because recent studies revealed that upregulation of several miRNAs induced by ketamine could be abolished by activation of GSK3 [27]. We are interested in the possibility that ketamine-induced miR-98-5p upregulation also involves GSK3, but further experiments are required to confirm that conjecture. Second, the downstream mechanisms of miR-98-5p in the pathology of depression and antidepressant effects of ketamine are also unclear. Since miR-98-5p has previously been reported to regulate PI3K/Akt signaling pathway [28], which is significantly involved in depression [29], and the involvement of PI3K/Akt in the antidepressant effect of ketamine has also been reported [30], we speculate that ketamine-induced upregulation of miR-98-5p may elicit the antidepressant effect through regulating PI3K/Akt pathway. To explore that possibility, we will investigate the role of PI3K/Akt pathway in the antidepressant effect of ketamine-induced miR-98-5p upregulation in our future work. Third, this study adopted agonists and antagonists to manipulate the miRNA expressions. It is also of interest to validate the results of this study by additional data obtained from other animal models.

In summary, we implicate miR-23a-5p, miR-98-5p, and miR-3968 in the pathogenesis of depression. Moreover, we uncover a novel role of miR-98-5p in ketamine’s antidepressant effect. However, the precise mechanisms about how ketamine regulates miR-98-5p expression and how miR-98-5p affects the progression of depression still require future exploration.

## Supplementary information

Supplementary material
