# Supplementary material for: miR-98-5p plays a critical role in depression and antidepressant effect of ketamine
Source: Transl Psychiatry. 2021 Sep 3;11:454. doi: 10.1038/s41398-021-01588-0 (PMC8417029; doi:10.1038/s41398-021-01588-0)

*Supplementary material*

**miR-98-5p plays a key role in the antidepressant-like effect of ketamine in rats**

Chaoli Huang^1,2#^, Yuanyuan Wang^1#^, Zifeng Wu^1^, Jiali Xu^1^, Ling Zhou^1^, Ling Yang^3^, Guiquan Chen^2^, Cunming Liu^1^, Chun Yang^1^

^1^Department of Anesthesiology and Perioperative Medicine, The First Affiliated Hospital of Nanjing Medical University, Nanjing 210029, China.

^2^State Key Laboratory of Pharmaceutical Biotechnology, Model Animal Research Center, Nanjing University, Nanjing 210061, China.

^3^Department of Cardiology, The Third Affiliated Hospital of Soochow University, Changzhou 213003, China.

#These authors contributed equally to this work.

Corresponding authors:

Dr. Chun Yang, Department of Anesthesiology and Perioperative Medicine, The First Affiliated Hospital of Nanjing Medical University, 300 Guangzhou Road, Nanjing 210029, China. Email: chunyang@njmu.edu.cn.

Dr. Cunming Liu, Department of Anesthesiology and Perioperative Medicine, The First Affiliated Hospital of Nanjing Medical University, 300 Guangzhou Road, Nanjing 210029, China. Email: cunmingliu@njmu.edu.cn.

**Figure s1**. Efficiency of antagomiRNA-23a-5p/98-5p/3968. **(a)** The schedule of antagomiRNA-23a-5p/98-5p/3968 transfections. **(b-d)** Real-time qPCR was performed to assay **t**he expression of miRNA-23a-5p/98-5p/3968 after antagomiRNA-23a-5p/98-5p/3968 transfections. Data are shown as mean ± SEM (n = 8). ***, *P* < 0.001.


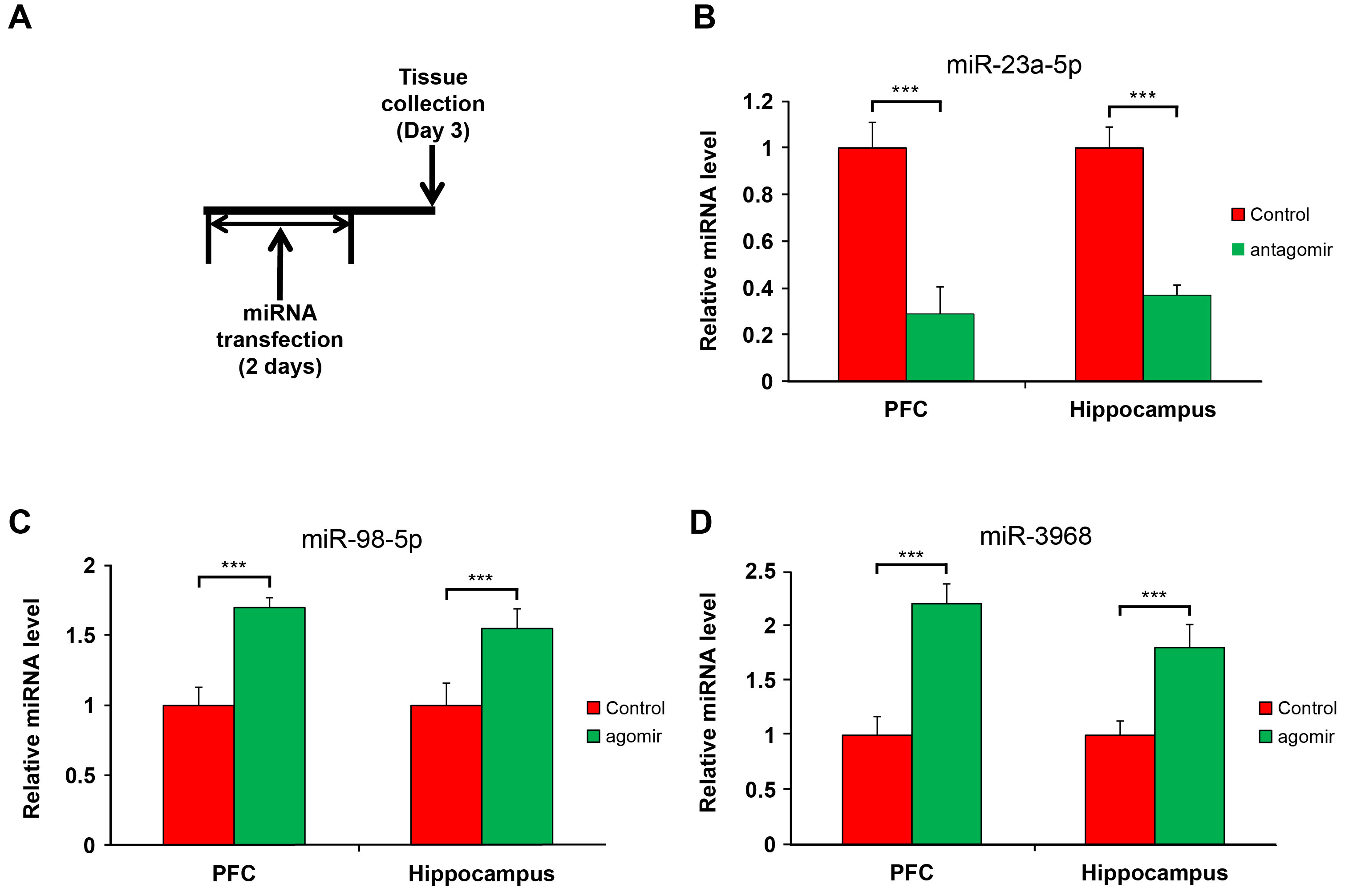

Supplement: Supplementary file 1 — Supplementary material [file 41398_2021_1588_MOESM1_ESM.docx]
